# Association between Nutritional Status with Spontaneous Abortion

**DOI:** 10.22074/ijfs.2016.4577

**Published:** 2016-11-01

**Authors:** Rahimeh Ahmadi, Saeideh Ziaei, Sosan Parsay

**Affiliations:** Department of Midwifery and Reproductive Health, Faculty of Medical Science, Tarbiat Modares University, Tehran, Iran

**Keywords:** Abortion, Nutrition, Pregnancy

## Abstract

**Background:**

Spontaneous abortion is the most common adverse pregnancy outcome. We aimed to investigate a possible link between nutrient deficiencies and the risk of spontaneous abortion.

**Materials and Methods:**

This case-control study included the case group (n=331) experiencing a spontaneous abortion before 14 weeks of pregnancy and the control group (n=331) who were healthy pregnant women over 14 weeks of pregnancy. The participants filled out Food Frequency Questionnaire (FFQ), in which they reported their frequency of consumption for a given serving of each food item during the past three months, on a daily, weekly or monthly basis. The reported frequency for each food item was converted to a daily intake. Then, consumption of nutrients was compared between the two groups.

**Results:**

There are significant differences between the two groups regarding consumed servings/day of vegetables, bread and cereal, meat, poultry, fish, eggs, beans, fats, oils and dairy products (P=0.012, P<0.001, P=0.004, P<0.001, P=0.019, respectively). There are significant differences between the two groups in all micronutrient including folic acid, iron, vitamin C, vitamin B6, vitamin B12 and zinc (P<0.001).

**Conclusion:**

Poor nutrientions may be correlated with increased risk of spontaneous abortion.

## Introduction

Spontaneous abortion is the most common adverse pregnancy outcomes occuring in approximately between 12 and 15% of clinically recognized pregnancies (1). The main reasons of early pregnancy loss are still unknown. Although a number of spontaneous abortions are caused by chromosomal abnormalities, maternal factors including nutritional status, also may contribute to this occurrence. The presence of appreciable amount of folic acid and vitamins has been reported to be essential for normal embryogenesis (1-3). There is reliable evidence indicating that maternal micronutrient status contributes to pregnancy outcome (4-9). A possible link has been suggested between nutrient deficiencies and reproductive risk factors. Interest has focused mainly on the risk of malformations. An association between intake of micronutrients, such as folic acid and zinc, magnesium and iron, and pregnancy outcome has been investigated (1-9).

In addition, reduced consumption of animal fats, carotene and proteins have been associated with the risk of hydatiform mole (10). Although the effects of maternal nutrition on fetal development and birth outcomes have been obviously demonstrated in animal studies, the findings of studies in humans inconsistent.

Due to influences of health eating habits on early phases of conception and pregnancy, we aimed to explore the association between nutrient deficiencies and the risk of spontaneous abortion.

## Materials and Methods

This case-control study was carried out in Tehran-Iran. The case group (n=331) experienced a spontaneous abortion before 14 weeks of pregnancy, while the control group (n=331) were healthy pregnant women over 14 weeks of pregnancy. Cluster sampling was used for selection of participations. In this method, ten regions were picked out randomly from 22 regions of Tehran, one public hospital was selected randomly from each picked region, and 331 cases was recruited randomly from these ten hospitals. Each case was assigned one control from these ten hospitals matched on maternal age, duration from last delivery in multiparous women, body mass index (BMI), occupation, and educational status.

Inclusion criteria were as follow: i. Singleton pregnancy, ii. 18-35 years of age, iii. No history of chronic diseases, such as diabetes, hypertension, cardiovascular diseases and thyroid dysfunction, iv. No history of any congenital or karyotypic abnormalities in the woman, her husband or one of the immediate relatives, v. Pregnancy without use of assisted reproductive technology (ART), vi. No vaginal bleeding in the first trimester of pregnancy in the control group, vii. Lack of fetal malformations in the current pregnancy, viii. No smoking during pregnancy.

The study was approved by the Ethical Committee of Tarbiat Modares University and a written informed consent was obtained from the participants.

At enrollment, the interviewers administered a questionnaire to the women to collect baseline information on socio demographic, and obstetrics history. Then, case group filled out dietary information after abortion in the hospital while the control group completed the same questionnaire.

### Dietary assessment

The assessment of dietary intake was performed using Food Frequency Questionnaire (FFQ) which was previously validated on the adult population of the city of Tehran, Iran (11). This questionnaire assesses 168 food items consumed in the preceding three months. This version of FFQ consists of a list of foods with a standard serving size commonly consumed by Iranian. For validity and reliability of FFQ, Mirmiran et al. (11) compared the dietary data collected monthly by means of twelve 24 hour dietary/recalls (24hDR) and biochemical markers with data collected from FFQ. The exact agreement rate was reasonable.

The participants were taught the food portion sizes using food photographs and measuring containers and given instructions for recording their dietary intake by a registered dietitian. The following food items were evaluated: fruits, vegetables, breads and cereals, dairy products, meat, poultry, fish, eggs, dry beans, and fats, oils, sweet. Due to presence of fats in above mentioned groups, dietary intake was calculated based on following information: 8 g fat in each ounce of sheep; 3 g fat in each ounces of veal meat; 1 g fat in each portion of bread and cereal; 5 g fat in each portion of low fat milk; 8 g fat in each portion of high fat milk .These amounts of fats were then added to the fat and oil group.

Participants were asked to report their frequency of consumption of a given serving size of each food item during the past three months, on a daily, weekly or monthly basis. The reported frequency for each food item was converted to a daily intake. Portion sizes of consumed food were then converted to gram using household measures. Also, the number of the women who consumed different numbers of food serving portion was compared between the two groups.

For assessment of micronutrients intake Mosbys Nutria Trace Nutrition Analysis Software was used.

### Statistical analysis

The sample size was estimated based on a pilot study, considering a power of 80% and alpha of 5%.

Overall comparisons between two groups were performed using t test or ϰ^2^ test. We computed the odds ratio (OR), as estimators of risks of spontaneous abortion, for the various parameters, with an approximate 95% confidence interval (CI). All statistical tests were two sided tests and were performed using a 5% significance level. The Statistical Package for the Social Sciences (SPSS, SPSS Inc, USA) version 20 was used for all statistical analysis.

## Results

Table 1 shows the characteristics of the cases and controls. There are no significant differences between the two groups regarding maternal age, duration from last delivery in multiparous women, BMI, occupation, and educational status.

There are significant differences between the two groups regarding consumed servings/day of vegetables, bread and cereal, meat, poultry, fish, eggs, dry beans, nuts, fats, oils, sweet and dairy products (P=0.012, P<0.001, P=0.004, P<0.001, P0.019, respectively) (Table 2).

**Table 1:** Comparison of demographics and obstetric characteristics between women with and without clinical spontaneous abortion


Variables	Case group (n=331)	Control group (n=331)	P value

Maternal age (Y)^*^	27.79 ± 5.30	27.31 ± 4.37	N.S
Duration from last delivery(months)^*^	41.78 ± 50.31	49.76 ± 45.97	N.S
BMI^*^	24.95 ± 6.72	24.25 ± 4.63	N.S
Occupation^**^			N.S
Housewife	293 (88.5)	308 (93.1)	
Employed	38 (11.5)	23 (6.9)	
Educational status^**^			N.S
Lower than university	267 (80.7)	275 (83.1)	
University	64 (19.3)	56 (16.9)	


^*^; Values are given as mean ± SD using Student’s t test, ^**^; Values are given as number (%) using Chi-squared test (χ^2^), N.S; Not significant, and BMI; Body mass index.

**Table 2:** Comparison of daily intake of food items between women with and without clinical spontaneous abortion. Values are given as number (%) ϰ^2^ test


Food items, portion per day	Case group (n=331)	Control group (n=331)	P value

Vegetables			0.012
(<3 parts)	282 (85.2)	253 (76.4)	
(3-5 parts)	49 (14.8)	77 (23.3)	
(>5 parts)	0 (0)	1 (0.3)	
Fruits			0.055
(<2 parts)	152 (45.9)	122 (36.9)	
(2-4 parts)	169 (51.1)	195 (58.9)	
(>4 parts)	10 (3)	14 (4.2)	
Breads and cereals			<0.001
(<6 parts)	129 (39)	88 (26.6)	
(6-11 parts)	199 (60.1)	208 (62.8)	
(>11 parts)	3 (0.9)	35 (10.6)	
Meat and beans			0.004
(<2parts)	268 (81.0)	232 (70.1)	
(2-3 parts)	63 (19.0)	98 (29.6)	
(>3 parts)	0 (0)	1 (0.3)	
Dairy products			<0.001
(<2 parts)	173 (52.3)	120 (36.3)	
(2-3 parts)	151 (45.6)	190 (57.4)	
(>3 parts)	7 (2.1)	21 (6.3)	
Fats and oils			0.019
(<55 g)	77 (23.3)	50 (15.1)	
(55-66 g)	113 (34.1)	114 (34.4)	
(>66 g)	141 (42.6)	167 (50.5)	


In addition, there are significant differences between the two groups in terms of all micronutrient factors including folic acid, iron, vitamin C, vitamin B6, vitamin B12 and zinc (Table 3).

**Table 3:** Comparison of daily intake of micronutrients between women with and without clinical spontaneous abortion*


Variables	Case group (n=331)	Control group (n=331)

Folic acid(µg)	416.09 ± 94.36	526.49 ± 73.41
Fe(mg)	19.29 ± 4.83	22.16 ± 4.03
Vitamin C (mg)	75.63 ± 7.62	78.06 ± 5.91
Vitamin B6 (mg)	1.12 ± 0.46	1.55 ± 0.33
Vitamin B12 (µg)	2.10 ± 0.39	2.32 ± 0.25
Zn (mg)	8.06 ± 1.50	9.93 ± 0.87


Values are given as mean ± SD by the independent t student test. *; P<0.001.

Table 4 depicts the OR and the 95% CI of logistic regression models for spontaneous abortion and consumption of micronutrients. For the women consuming the micronutrients OR of spontaneous abortion was significant in acid folic (0.986, 0.984-0.988), iron (0.868, 0.837-0.900), vitamin C (0.949, 0.928-0.971), vitamin B6 (0.096, 0.064-0.144), vitamin B12 (0.129, 0.076-0.218), and zinc (0.288, 0.237-0.349).

**Table 4:** Logistic regression models of the association between micronutrients and spontaneous abortion*


Variables	OR (95% CI)

Folic acid	0.986 (0.984-0.988)
Fe	0.868 (0.837-0.900)
Vitamin C	0.949 (0.928-0.971)
Vitamin B6	0.096 (0.064-0.144)
Vitamin B12	0.129 (0.076-0.218)
Zn	0.288 (0.237-0.349)


OR; Odds ratio, CI; Confidence interval, and *; P<0.001.

## Discussion

Our findings indicated that there are significant differences between the two groups in number of the women who consumed servings/day of vegetables, breads and cereals, meat, poultry, fish, eggs, dry beans, nuts, fats, oils, sweet and dairy products.

The incidence of pregnancy wastage is high in women from poor socio-economic groups. Maternal malnutrition is considered to be an important factor contributing to spontaneous abortions by way of altering the germ cell morphology; however, the relation between maternal nutrition and spontaneous abortion is complex and influenced by several biologic, socioeconomic, and lifestyle factors, which vary extremely in different populations (12, 13).

During pregnancy, there is an increased nutritional demand for both mother and fetus. Maternal under nutrition probably increases the risk of intrauterine death and abortion (14-17), possibly due to cellular dysfunction. Maternal nutritional deficiencies also cause serious damages on different stages of fetal development. A number of experimental animal studies and observational human studies have mentioned the consequences of malnutrition at the very earliest embryonic stages that affected fetal growth and birth outcomes (13, 18). Furthermore, evidence from animal studies have indicated that fetal growth is mostly affected by maternal under nutrition during the peri-implantation stage and the stage of rapid placental development (19, 20).

The maternal nutrition is more likely to be affected by socioeconomic and lifestyle factors in various ways. Socioeconomic status influences pregnancy dietary intake that results in multiple rather than single nutrient deficiencies. It has been also reported that cultural factors play a major role in maternal age at initiation of childbearing and interval between delivery (13, 16, 18, 21).

Karmer et al. (22) has stated that the countries with lowest rates of adverse birth outcomes had done so not through health care interventions but rather by reducing the prevalence of socioeconomic disadvantage and poverty.

According to the results of this study, the average of all micronutrients which was consumed by the case group was significantly lower than in the control group. The observed association in our study is in agreement with the findings of several previous studies. Optimal pregnancy outcomes are dependent upon the intake of adequate nutrients, and malnutrition results from inadequate diet, is synonymous with growth failure, especially during the rapid growth phases of fetus. It was recognized that poor pregnancy outcomes results not only from a deficiency of protein and macronutrient but also from inadequate intake of micronutrients that are vital during pregnancy (15-17).

Folate deficiency during pregnancy is also associated with numerous adverse pregnancy outcomes, including spontaneous abortions. A number of studies have indicated that suboptimal vitamin B6 status and elevated plasma total homocysteine concentrations a marker of poor folate or vitamin B12 status, may increase the risk of spontaneous abortion (1, 2, 6, 23, 24), although the association is still unknown. Elevated plasma total homocysteine concentrations are caused by genetic abnormalities or suboptimal folate, vitamin B12, and/or vitamin B6 status.

We also found lower consumption of vitamin C in the women with spontaneous abortion. In a report based on the women with recurrent abortion by Vural et al. (25), they have found that antioxidant elements including vitamin C decreased in case group in comparison with healthy pregnant women.

In the present study, women with spontaneous abortion had lower consumption of iron and zinc as compared to the control women. This is consistent with other studies, in which they have found an association between poor zinc status and adverse pregnancy outcomes (22, 26-29)

The present study had several limitations. A major limitation of this study was the self-report of dietary data and the lack of precise estimation of micronutrients and macronutrients consumption. Another limitation of our study was that neither embryos nor parents were karyotyped. Aneuploidy is a common cause of spontaneous abortion and that was not excluded in our study. The third limitation was that any hospital-based case-control study on abortion includes only women with spontaneous abortion requiring hospital admission, with the consequent exclusion of women with subclinical abortions or very early pregnancy losses.

A strength of the current study was dietary data were collected approximately at same gestational ages in cases and controls groups; meaning the case group was interviewed in hospital and the control group in prenatal clinics. Further, the differences observed could not be explained by some confounding factors because these parameters were matched as far as possible between the two groups.

## Conclusion

Despite potential limitations and difficulties in the interpretation, our findings illustrated that a diet poor in several nutrients may increase risk of spontaneous abortion.

## References

[1] Nelen WL, Blom HJ, Steegers EA, den Heijer M, Thomas CM, Eskes TK (2000). Hemocystein and folate levels as risk factors for recurrent pregnancy loss. Obstet Gynecol.

[2] Ronnenberg AG, Goldman MB, Chen D, Aitken IW, Willett WC, Selhab J (2002). Preconception folate and vitamin B status and clinical spontaneous abortion in Chinese women. Obstet Gynecol.

[3] Zetterberg H, Regland B, Palmer M, Rymo L, Zafiropoulos A, Arvanitis DA (2002). The transcobalamin codon 259 polymorphism influences the risk of human spontaneous abortion. Hum Reprod.

[4] Ziaei S, Janghorban R, Shariatdoust S, Faghihzadeh S (2008). The effects of iron supplementation on serum copper and zinc levels in pregnant women with high-normal hemoglobin. Int J Gynecol Obstet.

[5] Fall CH, Yajnik CS, Rao S, Davies AA, Brown N, Farrant HJ (2003). Micronutrients and fetal growth. J Nutr.

[6] de la Calle M, Usandizaga R, Sancha M, Magdaleno F, Herranz A, Cabrillo E (2003). Homocysteine, folic acid and Bgroup vitamins in obstetrics and gynecology. Eur J Obstet Gynecol Reprod Biol.

[7] Black RE (2001). Micronutrients in pregnancy. Br J Nutr.

[8] Rao S, Yajnik CS, Kanade A, Fall CH, Margetts BM, Jackson AA (2001). Intake of micronutrient-rich foods in rural Indian mothers is associated with the size of their bodies at birth: pune maternal nutrition study. J Nutr.

[9] Keen CL, Clegg MS, Hanna LA, Lanoue L, Rogers JM, Daston GP (2003). The plausibility of micronutrient deficiencies being a significant contributing factor to the occurrence of pregnancy complications. J Nutr.

[10] Di Cintio E, Parazzini F, Chatenoud L, Surace M, Benzi G, Zanconato G (2001). Dietary factors and risk of spontaneous abortion. Eur J of Obstet Gynecol Reprod Biol.

[11] Mirmiran P, Esfahani FH, Mehrabi Y, Hedayati M, Azizi F (2010). Reliability and relative validity of an FFQ for nutrient in the Tehran lipid and glucose study. Public Health Nutr.

[12] Ashworth CJ, Antipatis C (2001). Micronutrient programming of development throughout gestation. Reproduction.

[13] Villar J, Merialdi M, Gülmezoglu AM, Abalos E, Carroli G, Kulier R (2003). Nutritional interventions during pregnancy for the prevention or treatment of maternal morbidity and preterm delivery:an overview of randomized controlled trials. J Nutr.

[14] Abu-Saad K, Fraser D (2010). Maternal nutrition and birth outcome. Epidemiol Rev.

[15] Wu G, Bazer FW, Cudd TA, Meininger CJ, Spencer TE (2004). Maternal nutrition and fetal development. J Nutr.

[16] Maternal nutrition: new developments and implications (2000). Proceedings of a symposium.Paris, France, June 11-12, 1998. Am J Clin Nutr.

[17] Ramachandran P (2002). Maternal nutrition-effect on fetal growth and outcome of pregnancy. Nutr Rev.

[18] Scroll TO (2005). Iron status during pregnancy:setting the stage for mother and infant. Am J Clin Nutr.

[19] Nafee TM, Farrell WE, Carroll WD, Fryer AA, Ismail KM (2008). Epigenetic control of fetal gene expression. BJOG.

[20] Waterland RA, Michels KB (2007). Epigenetic epidemiology of the developmental origins hypothesis. Annu Rev Nutr.

[21] Allen LH (2000). Anemia and iron deficiency: effects on pregnancy outcome. Am J Clin Nutr.

[22] Kramer MS, Seguin L, Lydon J, Goulet L (2000). Socio-economic disparities in pregnancy outcome:why do the poor fare so poorly?. Paediatr Perinat Epidemiol.

[23] Coumans AB, Huijgens PC, Jacobs C, Schats R, de Vries JI, van Pampus MG (1999). Haemostatic and metabolic abnormalities in women with unexplained recurrent abortion. Hum Reprod.

[24] Ronnenberg AG, Venners SA, Xu X, Chen C, Wang L, Guang W (2007). Preconception B-vitamin and homocysteine status,conception and early pregnancy loss. Am J Epidemiol.

[25] Vural P, Akgül C, Yildirim A, Canbaz M (2000). Antioxidant defense in recurrent abortion. Clin Chim Acta.

[26] Abdul-Barry J, Al-Rubai SA, Qasim QA (2011). Study of oxidantantioxidant status in recurrent spontaneous abortion. ThiQar Medical Journal.

[27] Picco SJ, Anchordoquy JM, de Matos DG, Anchordoquy JP, Seoane A, Mattioli GA (2010). Effect of increasing zinc sulphate concentration during in vitro maturation of bovin oocytes. Theriogenology.

[28] Graham TW, Thurmond MC, Gershwin ME, Picanso JP, Garvey JS, Keen CL (1994). Serum zinc and copper concentrations in relation to spontaneous abortion in cows:implications for human fetal loss. J Reprod Fertil.

[29] Zare A, Saremi A, Hajhashemi M, Kardar GA, Moazzeni SM, Pourpak Z (2013). Correlation between serum zinc levels and successful immunotherapy in recurrent spontaneous abortions. J Hum Reprod Sci.

